# Алемтузумаб-индуцированная болезнь Грейвса

**DOI:** 10.14341/probl13238

**Published:** 2023-06-30

**Authors:** М. С. Шеремета, М. О. Корчагина, Р. М. Гусейнова, Т. Е. Шмидт, К. С. Нижегородова, Н. Ю. Свириденко, Г. А. Мельниченко

**Affiliations:** Национальный медицинский исследовательский центр эндокринологии; Национальный медицинский исследовательский центр эндокринологии; Национальный медицинский исследовательский центр эндокринологии; Первый Московский государственный медицинский университет им. И.М. Сеченова (Сеченовский Университет); Национальный медицинский исследовательский центр эндокринологии; Национальный медицинский исследовательский центр эндокринологии; Национальный медицинский исследовательский центр эндокринологии

**Keywords:** рассеянный склероз, алемтузумаб, тиреотоксикоз, терапия радиоактивным йодом

## Abstract

Рассеянный склероз (РС) — это тяжелое хроническое аутоиммунное демиелинизирующее заболевание центральной нервной системы, опосредованное Th1/Th17-лимфоцитами, а также В-лимфоцитами, макрофагами и другими иммунными клетками. Часть пациентов с РС в качестве лечения получают алемтузумаб — моноклональное антитело против CD52+ клеток, относящееся к группе препаратов, изменяющих течение РС. Основной эффект алемтузумаба связан с изменением иммунного профиля, что может привести к развитию вторичного аутоиммунитета на фоне восстановления пула клеток иммунной системы. При этом наиболее часто в аутоиммунный процесс вовлекается щитовидная железа — чаще всего развивается болезнь Грейвса (БГ), за ней по частоте следует аутоиммунный тиреоидит.

Мы представляем клинический случай ведения пациентки с БГ, развившейся после курса лечения РС алемтузумабом. Пациентка была направлена к радиологу в отделение радионуклидной терапии ГНЦ РФ ФГБУ «НМИЦ эндокринологии» Минздрава России для проведения терапии радиоактивным йодом в связи с неэффективностью тиреостатической терапии БГ. Цель лечения достигнута через 2 месяца, инициирована терапия тиреоидными гормонами, на фоне чего наблюдается компенсация функции щитовидной железы.

## Abstract (en)

Multiple sclerosis (MS) is a severe chronic autoimmune demyelinating disease of the central nervous system, mediated by Th1/Th17 lymphocytes as well as B lymphocytes, macrophages and other immune cells. Some patients with MS are treated with alemtuzumab, a monoclonal antibody against CD52+ cells, which belongs to the disease-modifying therapies (DMTs). The main effect of alemtuzumab is related to changes in immune recruitment. Alemtuzumab therapy can induce secondary autoimmunity against the background of immune rebalancing. The thyroid gland is generally involved in the autoimmune process. Graves’ disease (GD) develops most often, followed by autoimmune thyroiditis.

We present a clinical case of a patient with GD developed after alemtuzumab therapy for MS. The patient was referred to a radiologist at the Department of Radionuclide Therapy of Endocrinology Research Centre for radioiodine therapy (RAIT) due to relapse of thyrotoxicosis after anti-thyroid drug therapy for GD. The goal of treatment was achieved in 2 months, thyroid hormone therapy was initiated, against the background of this, there was compensation of thyroid function.

## АКТУАЛЬНОСТЬ

Одним из наиболее распространенных аутоиммунных неврологических заболеваний является рассеянный склероз (РС) — хроническое воспалительное заболевание, характеризующееся демиелинизацией, вторичной дегенерацией аксонов в центральной нервной системе и неизменно приводящее к стойкой инвалидизации, нарушению функционирования и потере социальной активности [1]. Наиболее часто РС дебютирует в возрасте от 20 до 40 лет и становится основной причиной инвалидизации у молодых людей. Соотношение женщин и мужчин при РС составляет примерно 2:1 [2].

РС — мультифакториальное заболевание, этиология которого связана с вирусной инфекцией, генетической предрасположенностью, влиянием географического фактора (место проживания) [3–5].

Лечение РС включает терапию обострений, применение препаратов, изменяющих течение РС (ПИТРС), и симптоматическую терапию [6][7]. ПИТРС в основном предназначены для сокращения частоты обострений. Они используются при активном течении ремиттирующего РС, который характеризуется клиническими обострениями или наличием активности по данным магнитно-резонансной томографии (МРТ) [8]. Одним из ПИТРС является алемтузумаб — рекомбинантное гуманизированное IgG1-каппа моноклональное антитело, нацеленное на CD52 [9]. CD52 — это гликопротеин, который экспрессируется на поверхности более 95% Т- и В-клеток, моноцитов, части дендритных клеток, естественных киллеров и на некоторых других клетках иммунной системы. Связывание алемтузумаба с лимфоцитами вызывает их лизис и быструю, длительную элиминацию из циркуляторного русла.

Алемтузумаб может вызывать длительную ремиссию в результате короткого курса лечения РС. После истощения популяции лимфоцитов происходит их репопуляция, включая изменение количества, процентного соотношения и свойств некоторых субпопуляций лимфоцитов, увеличение количества субпопуляций регуляторных Т-лимфоцитов (Treg). Снижение количества циркулирующих В- и Т-лимфоцитов после терапии алемтузумабом и их последующая репопуляция снижают частоту рецидивов и прогрессирование инвалидизации при РС. Алемтузумаб также оказывает транзиторное влияние на клетки врожденного иммунитета, нейтрофилы, макрофаги и естественные киллеры [10][11].

Основным побочным эффектом препарата является развитие дополнительных аутоиммунных нарушений, возникающих, как правило, в течение 5 лет с момента лечения и с пиком заболеваемости на третий год после первого введения препарата [12][13]. Наиболее часто встречается аутоиммунное поражение щитовидной железы (ЩЖ) — до 41% случаев [11]. Другие аутоиммунные заболевания включают иммунную тромбоцитопению и нефропатию [14].

## КЛИНИЧЕСКИЙ СЛУЧАЙ

Пациентка К., 39 лет, прошла радиойодтерапию (РЙТ) в отделении радионуклидной терапии ГНЦ РФ ФГБУ «НМИЦ эндокринологии» Минздрава России (НМИЦ эндокринологии) по поводу диффузного токсического зоба на фоне лечения РС препаратом алемтузумаб.

Из анамнеза известно, что в 2012 г. обратилась к неврологу по месту жительства с жалобами на асимметрию лица, головокружение. По результатам комплексного обследования установлен диагноз РС, высокоактивное ремиттирующее течение с двусторонней пирамидной симптоматикой, легким вестибулярным синдромом исходно EDSS (Expanded Disability Status Scale, расширенная шкала степени инвалидизации) 2,5 балла. С 2013 по 2018 гг. в связи с частыми обострениями пациентка получала терапию интерфероном бета-1b, на фоне которой обострения РС фиксировались до 3 раз в год. В 2018 г. в связи с неэффективностью терапии и выраженной декомпенсацией заболевания (нарастанием EDSS до 6,5 балла), а также прогрессированием радиологически активных очагов по данным МРТ головного мозга был назначен алемтузумаб.

Первый курс лечения проведен в ноябре 2018 г. и составил 5 внутривенных инфузий по 12 мг в сутки. Через 1 год пациентка прошла второй курс — 3 внутривенные инфузии по 12 мг в сутки. Нежелательных явлений не наблюдалось. На этом фоне достигнута стабилизация течения РС. При последующих МРТ головного мозга в 2018–2022 гг. сохранялись множественные очаговые изменения без признаков активности и накопления контрастного вещества, отрицательной динамики не было. В мае 2021 г. через 6 мес после второго курса алемтузумаба пациентка впервые отметила симптомы, характерные для тиреотоксикоза, — учащенный пульс до 110 ударов в минуту в покое, потливость, слабость, тревожность, снижение массы тела на 7 кг за 2 мес.

При дообследовании у эндокринолога выявлен манифестный тиреотоксикоз, повышение уровня антител к рецепторам тиреотропного гормона (АТ-рТТГ) (табл. 1). Был установлен диагноз — болезнь Грейвса (БГ). Инициирована тиреостатическая терапия тиамазолом в начальной дозе 30 мг в сутки с последующим снижением. На фоне консервативной терапии достигнуть ремиссии не удалось, при постепенном снижении дозы тиамазола до 5 мг в сутки отмечено усиление тиреотоксикоза.

**Table:** Таблица 1. Динамика лабораторных показателейTable 1. Dynamics of laboratory parameters Примечание. ТТГ — тиреотропный гормон; св. Т4 — свободный тироксин; св. Т3 — свободный трийодтиронин; АТ-рТТГ — антитела к рецепторам тиреотропного гормона.

Дата исследования	Показатель, единицы измерения	Значение	Референсный интервал
Октябрь 2018 До начала лечения алемтузумабом	ТТГ, мМЕ/л св.Т4, пмоль/л св.Т3, пмоль/л	1,48 12,05 3,5	0,4–4,6 11–22 3,8–7,3
Май 2021 Через 18 мес после 2-го курса алемтузумаба	ТТГ, мМЕ/л св.Т4, пмоль/л св.Т3, пмоль/л АТ-рТТГ, Ед/л	0,024 21,3 8,6 19,07	0,4–4,6 11–22 3,8–7,3 0–1,5
Апрель 2022 на фоне тиреостатической терапии (11 мес)	ТТГ, мМЕ/л св.Т4, пмоль/л св.Т3, пмоль/л	0,003 13,1 4,5	0,4–4,6 11–22 3,8–7,3
Сентябрь 2022 После РЙТ	ТТГ, мМЕ/мл	10,69	0,4–4,6

По данным ультразвукового исследования (УЗИ) за период заболевания отмечено увеличение объема ЩЖ с 12,3 до 24,7 см3 (норма для женщин до 18 см3), структура паренхимы ЩЖ неоднородная за счет диффузных изменений по типу чередования гипо- и гиперэхогенных участков, васкуляризация значимо повышена, узловые образования не визуализируются. По данным сцинтиграфии ЩЖ с 99mТс-пертехнетатом распределение радиофармацевтического препарата (РФП) равномерное (правая доля 51%, левая доля 49%), индекс захвата 99mTc-пертехнетата повышен до 6,4% (диапазон референсных значений от 0,7 до 1,8%), накопление в левой и правой долях диффузное, повышенное (рис. 1).

**Figure:**
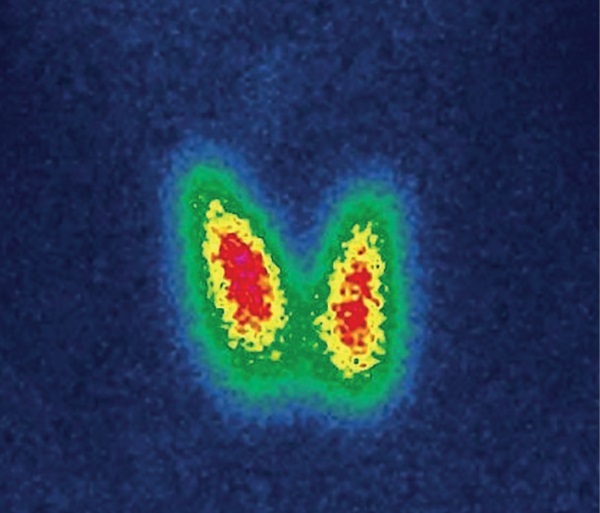


С целью исключения эндокринной офтальмопатии (ЭОП) пациентке проведено комплексное офтальмологическое обследование. Признаков ЭОП не выявлено.

Учитывая длительность тиреотоксикоза, отсутствие ремиссии спустя 14 мес тиреостатической терапии и низкую ожидаемую эффективность консервативной терапии в дальнейшем, а также риск прогрессирования РС, рекомендовано радикальное лечение БГ. Принимая во внимание данные УЗИ (объем ЩЖ 24,7 см3), сцинтиграфии (повышение индекса захвата 99mТс-пертехнетата до 6,4%), желание пациентки, методом лечения стала РЙТ. С целью усиления захвата 131I и повышения эффективности РЙТ за 7 дней до введения 131I отменена терапия тиамазолом, исключено воздействие лекарственных йодсодержащих препаратов и контрастных веществ. В июле 2022 г. пациентка была госпитализирована в отделение радионуклидной терапии НМИЦ эндокринологии, проведена РЙТ активностью 610 МБк. В течение 2 мес после РЙТ тиреотоксикоз полностью купирован, развился постлучевой гипотиреоз (см. табл. 1). Под наблюдением врача-эндокринолога по месту жительства пациентке назначена терапия тиреоидными гормонами (левотироксин в дозе 75 мкг в сутки). В настоящее время наблюдается компенсация функции ЩЖ.

## ОБСУЖДЕНИЕ

Концепция патогенеза РС включает выделение начальной воспалительной фазы, проявляющейся демиелинизацией проводников центральной нервной системы, которая затем сменяется фазой нейродегенерации, приводящей к повреждению самих осевых цилиндров (аксонов) [15–17]. В результате каскада иммунологических и биохимических нарушений развивается повреждение миелина и олигодендроцитов. На более поздних этапах патологического процесса активируются неспецифические механизмы: фагоцитоз поврежденных структур и пролиферация глиальных элементов. Иммуновоспалительные изменения и демиелинизирующее поражение сопровождаются гибелью аксонов, которая наблюдается уже на ранних стадиях заболевания [18]. Именно аксональное повреждение отвечает за развитие необратимого неврологического дефицита и трансформацию ремиттирующего течения с лабильностью симптомов во вторично-прогрессирующее [19].

Для РС характерна разнообразная клиническая симптоматика, связанная с поражением различных участков головного и спинного мозга. Выделяют три типа течения РС: ремиттирующий, первичный прогрессирующий и вторичный прогрессирующий. В подавляющем большинстве случаев РС дебютирует ремиттирующим типом течения (РРС), который характеризуется наличием обострений с появлением новых симптомов и последующими ремиссиями [20]. Рецидивы при РРС возникают из-за очагов демиелинизации, развивающихся в течение 24 ч и сохраняющихся в течение нескольких дней или недель. Впоследствии, как правило, наблюдается полное или чаще неполное восстановление [6]. Однако серия обострений с неизбежностью приводит к накоплению инвалидизации.

Клинические исследования показывают, что заболевания ЩЖ — наиболее частые нежелательные явления (НЯ) после терапии алемтузумабом. Дисфункция ЩЖ может варьировать от легкого, самолимитирующегося тиреоидита до тяжелого тиреотоксикоза, и именно изменения в адаптивном иммунном ответе лежат в основе аутореактивности и дальнейшего развития аутоиммунных заболеваний.

У пациентов могут обнаруживаться АТ-рТТГ и/или антитела к тиреоидной пероксидазе (АТ-ТПО) с клиническими проявлениями или без них. Отмечено, что риск развития НЯ со стороны ЩЖ повышен у пациентов с положительными АТ-ТПО, что также необходимо учитывать при назначении алемтузумаба [21]. БГ является наиболее распространенным вариантом нарушения функции ЩЖ после терапии алемтузумабом [22]. На ее долю приходится более половины всех случаев аутоиммунитета [15]. Это объясняется тем, что аутоиммунитет чаще связан с синтезом аутоантител, а не с деструктивными Th1-опосредованными реакциями. Алемтузумаб приводит к истощению CD4+ и CD8+ Т-клеток, а также CD19+ В-клеток (рис. 2). При этом после истощения последних происходит выраженная гиперпопуляция незрелых В-клеток (увеличение на 180%) с их дальнейшем преобразованием в зрелые В-клетки. В-клетки восстанавливаются в среднем через 3 мес, что гораздо быстрее, чем Т-клетки. Количество CD8+ Т-клеток восстанавливается через 20 мес, CD4+ Т-клеток, включая Treg, — через 35 мес. Как известно, Treg выполняют супрессорную функцию, и их главная задача — предотвращение развития аутоиммунных заболеваний. Вызванная алемтузумабом лимфоцитопения сопровождается гомеостатическим ростом Т-клеток, который стимулируется комплексом Т-клеточный рецептор–пептид. Этот процесс приводит к появлению олигоклональной популяции клеток, склонных к аутореактивности. Treg не способны предотвратить аутоиммунные реакции отчасти от того, что Т-клетки подвергаются более быстрому гомеостатическому росту. С восстановлением пула Т-хелперов усиливаются процессы дифференцировки В-лимфоцитов в плазматические клетки и В-клетки памяти. Считается, что на этом фоне отсутствие адекватного контроля со стороны Treg становится причиной высокой распространенности аутоиммунных реакций после лечения алемтузумабом. После введения алемтузумаба и элиминации клеток иммунной системы из кровотока в кровь вновь поступает большое количество незрелых В-клеток из костного мозга, что в отсутствие эффективной Т-клеточной регуляции с помощью Treg и регуляторных/супрессорных клеток CD8+ может привести к появлению и сохранению аутореактивных В-клеток [23].

**Figure:**
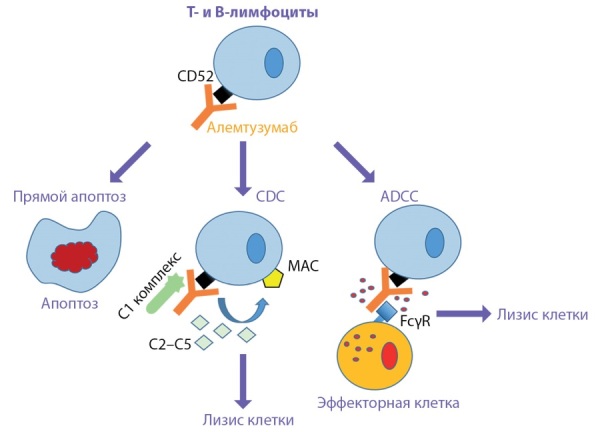
Рисунок 2. Механизм действия алемтузумаба.Примечание. Алемтузумаб взаимодействует с CD52 и вызывает элиминацию B- и T-лимфоцитов из кровяного русла путем прямого апоптоза или индуцируя лизис лимфоцитов. Лизис опосредован системой комплемента и антителозависимым клеточным цитотоксическим эффектом.Сокращения: CDC — комплемент-зависимая цитотоксичность; ADCC — антителозависимая клеточная цитотоксичность; MAС — мембраноатакующий комплекс (С5b-С9), С1–С5 — компоненты системы комплемента; FcγR — Fc-гамма рецептор.Figure 2. Mechanism of action of alemtuzumab

Отмечено, что БГ, вызванная алемтузумабом, характеризуется высокой частотой ремиссий, как самопроизвольных, так и после курса тиреостатиков, а также спонтанным переходом к гипотиреозу в результате изменения преобладающих АТ-рТТГ — от стимулирующих к блокирующим [17]. Рецептор к ТТГ является основным аутоантигеном при БГ. АТ-рТТГ могут быть стимулирующими, блокирующими и нейтральными. У пациентов с БГ АТ-рТТГ в основном обладают стимулирующей активностью, что приводит к тиреотоксикозу и формированию диффузного токсического зоба. АТ-рТТГ связывают только естественно конформированный рТТГ и вызывают выработку циклического аденозинмонофосфата, пролиферацию клеток ЩЖ, синтез и секрецию йодтиронинов. Аутоиммунитет при БГ представлен рТТГ-реактивными В-клетками, которые избегают уничтожения и представляют тиреоидный аутоантиген Т-клеткам [24]. Важная роль отводится Т-хелперам (Th) 1. В активной фазе заболевания они секретируют хемокины Th1, которые взаимодействуют со своими рецепторами CXCR3 [25]. Нарушение активности Т-reg, пролиферация аутореактивных Т- и В-клеток, усиленная презентация рТТГ и ряд других событий способствуют развитию и закреплению аутоиммунного каскада при БГ [26].

Около 15% пациентов с БГ, индуцированной приемом алемтузумаба, имеют нестабильное течение [27]. БГ, ассоциированная с применением алемтузумаба, может протекать менее агрессивно. Исследование CAMMS223 показало, что у 22% пациентов, получавших алемтузумаб, развилась БГ. Среди них в 78% случаев наблюдалась либо спонтанная ремиссия, либо ремиссия после антитиреоидной терапии [28]. Похожие наблюдения были сделаны в исследовании CARE-MS II, где во всех случаях БГ применялись антитиреоидные препараты, и только двум пациентам потребовалось радикальное лечение [13].

Предполагается, что индивидуальный риск развития вторичного аутоиммунитета увеличивается под влиянием курения в 3 раза, семейного анамнеза — в 7 раз. При этом курение, семейный анамнез заболеваний ЩЖ и высокий титр тиреоидных антител были связаны с повышенным риском развития более тяжелой формы заболевания. Роль пола неясна: исследования показывают отсутствие различий или увеличение риска в 2 раза у женщин в сравнении с мужчинами [29]. В представленном клиническом случае пациентка не курила и не имела семейного анамнеза заболеваний ЩЖ, однако у нее был высокий титр АТ-ТПО. С целью исследования функции ЩЖ определение уровня ТТГ в сыворотке крови должно быть проведено до начала лечения алемтузумабом и далее каждые 3 мес (факультативно может быть исследован уровень свТ4). Мониторинг функции ЩЖ рекомендовано продолжать до 48 мес после последней инфузии алемтузумаба (рис. 3). После этого периода определение уровня ТТГ следует проводить на основании клинической картины, свидетельствующей о дисфункции ЩЖ [30–32]. При развитии дисфункции ЩЖ пациент должен быть немедленно направлен к эндокринологу.

**Figure:**
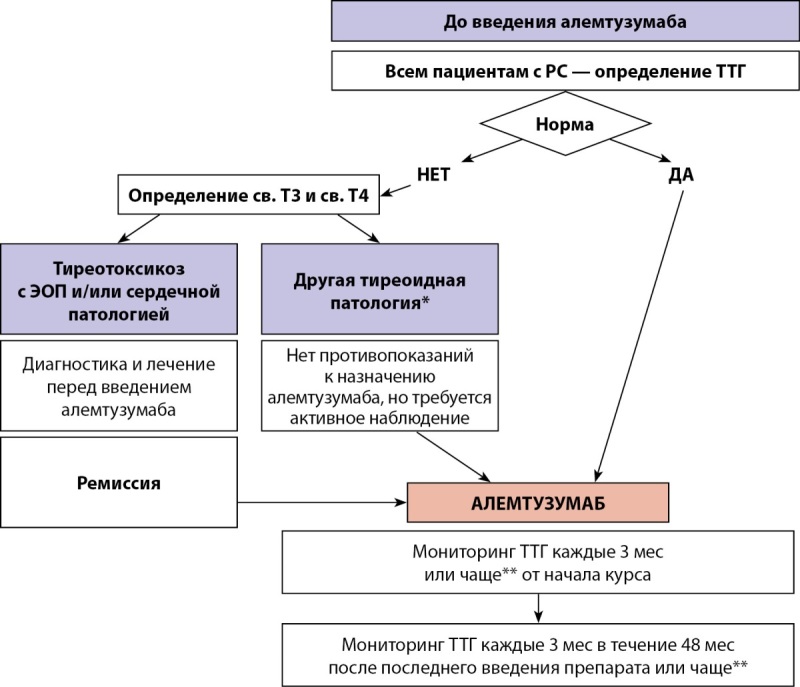
Рисунок 3. Алгоритм ведения пациента с РС, получающего алемтузумаб.Примечание.*Тиреотоксикоз без признаков ЭОП и/или сердечной патологии, гипотиреоз, аутоиммунный тиреоидит. ** При первом появлении симптомов тиреоидной патологии.Figure 3. Algorithm for managing a patient with MS receiving alemtuzumab

## ЗАКЛЮЧЕНИЕ

Клинически доказано, что у пациентов, получавших алемтузумаб по поводу РС, возможно ожидать развития дисфункции ЩЖ, и БГ — наиболее часто встречаемая аутоиммунная патология, за ней следует аутоиммунный тиреоидит. Особенностью течения заболеваний ЩЖ у данной группы пациентов является частый спонтанный переход тиреотоксикоза в гипотиреоз, и наоборот. Более того, РС — дополнительный фактор риска развития вторичных аутоиммунных нарушений.

Описанный нами клинический случай демонстрирует необходимость ранней диагностики заболевания и важность информирования как пациентов, так и клиницистов о возможных побочных эффектах алемтузумаба, а также о симптомах тиреоидной патологии. Вторичная аутоиммунная патология, вызванная алемтузумабом, может повлиять на течение РС, поэтому необходимы ее своевременная диагностика и скорейшее лечение.

## ДОПОЛНИТЕЛЬНАЯ ИНФОРМАЦИЯ

Источники финансирования. Данная работа выполнена в соответствии с планом государственного задания. Регистрационный номер 123021000041-6.

Конфликт интересов. Авторы декларируют отсутствие явных и потенциальных конфликтов интересов, связанных с содержанием настоящей статьи.

Участие авторов. Все авторы внесли одинаковый вклад в написание работы, одобрили финальную версию статьи перед публикацией, выразили согласие нести ответственность за все аспекты работы, подразумевающую надлежащее изучение и решение вопросов, связанных с точностью или добросовестностью любой части работы.

Согласие пациента. Пациентка добровольно подписала информированное согласие на публикацию персональной медицинской информации в обезличенной форме в журнале «Проблемы эндокринологии».
